# Evaluation of impact of anti-idursulfase antibodies during long-term idursulfase enzyme replacement therapy in mucopolysaccharidosis II patients

**DOI:** 10.1016/j.ymgmr.2017.01.014

**Published:** 2017-02-21

**Authors:** R. Giugliani, P. Harmatz, S.A. Jones, N.J. Mendelsohn, A. Vellodi, Y. Qiu, C.J. Hendriksz, S. Vijayaraghavan, D.A.H. Whiteman, A. Pano

**Affiliations:** a Department of Genetics/UFRGS, Medical Genetics Service/HCPA, INAGEMP, Porto Alegre, Brazil; b UCSF Benioff Children's Hospital Oakland, Oakland, CA, USA; c Manchester Centre for Genomic Medicine, St Mary's Hospital, MAHSC, Manchester, UK; d Genomic Medicine Program, Children's Hospitals and Clinics of Minnesota, and Division of Medical Genetics, Department of Pediatrics, University of Minnesota, Minneapolis, MN, USA; e Metabolic Medicine Unit, Great Ormond Street Hospital for Children NHS Foundation Trust, London, UK; f Shire, Lexington, MA, USA; g Department of Paediatrics and Child Health, University of Pretoria, Steve Biko Academic Unit, Pretoria, South Africa; h Department of Clinical Inherited Metabolic Disorders, Birmingham Children's Hospital NHS Foundation Trust, Birmingham, UK

**Keywords:** Neutralizing antibodies, Idursulfase, Hunter syndrome, Enzyme replacement therapy, Cognitive impairment, Immunogenicity, Glycosaminoglycans

## Abstract

**Objectives:**

This 109-week, nonrandomized, observational study of mucopolysaccharidosis II (MPS II) patients already enrolled in the Hunter Outcome Survey (HOS) (NCT00882921), assessed the long-term immunogenicity of idursulfase, and examined the effect of idursulfase-specific antibody generation on treatment safety (via infusion-related adverse events [IRAEs]) and pharmacodynamics (via urinary glycosaminoglycans [uGAGs]).

**Methods:**

Male patients ≥ 5 years, enrolled in HOS regardless of idursulfase treatment status were eligible. Blood/urine samples for anti-idursulfase antibody testing and uGAG measurement were collected every 12 weeks.

**Results:**

Due to difficulties in enrolling treatment-naïve patients, data collection was limited to 26 enrolled patients of 100 planned patients (aged 5.1–35.5 years) all of whom were non-naïve to treatment. Fifteen (58%) patients completed the study. There were 11/26 (42%) seropositive patients at baseline (Ab +), and 2/26 (8%) others developed intermittent seropositivity by Week 13. A total of 9/26 patients (35%) had ≥ 1 sample positive for neutralizing antibodies. Baseline uGAG levels were low due to prior idursulfase treatment and did not change appreciably thereafter. Ab + patients had persistently higher uGAG levels at entry and throughout the study than Ab − patients. Nine of 26 (34%) patients reported IRAEs. Ab + patients appeared to have a higher risk of developing IRAEs than Ab − patients. However, the relative risk was not statistically significant and decreased after adjustment for age.

**Conclusions:**

50% of study patients developed idursulfase antibodies. Notably Ab + patients had persistently higher average uGAG levels. A clear association between IRAEs and antibodies was not established.

## 1 Introduction

Hunter syndrome (mucopolysaccharidosis II [MPS II]) is characterized by a deficiency in iduronate-2-sulfatase, a key enzyme in the catabolism of glycosaminoglycans (GAGs) [1]. Individuals display significant morbidity and early mortality, with approximately two-thirds experiencing progressive cognitive impairment (severe phenotype) and approximately one-third of patients demonstrating intact cognition (attenuated phenotype) [2].

Recombinant iduronate-2-sulfatase (idursulfase, Elaprase®, Shire, Lexington, MA, USA) is approved in many countries for enzyme replacement therapy (ERT) of patients with MPS II [3].

While studies of idursulfase have consistently demonstrated safety and efficacy, roughly 50% of patients produce idursulfase-specific serum immunoglobulin G (IgG) antibodies [3], [4], [5].

This 109-week, nonrandomized, observational study of Hunter syndrome patients was a sub-study within the Hunter Outcome Survey (HOS), a global registry of patients with Hunter syndrome, established to enhance understanding of Hunter syndrome natural history and to monitor the long-term safety and effectiveness of idursulfase in a large patient cohort [6]. The study monitored anti-idursulfase antibody development in Hunter syndrome patients after long-term idursulfase ERT and was designed to include patients in HOS who had previously received idursulfase, as well as treatment-naïve HOS patients who had planned to begin idursulfase treatment within 30 days of enrollment in this study. The primary study objective was to evaluate the effect of anti-idursulfase IgG, IgM, and IgE antibodies on idursulfase safety (as measured by infusion-related adverse events [IRAEs]) between patients who develop anti-idursulfase antibodies and patients who do not after long-term idursulfase ERT (NCT00882921). The secondary study objective was to evaluate the effects of anti-idursulfase IgG antibodies on idursulfase pharmacodynamics (as measured by urinary glycosaminoglycan [uGAG] levels).

## 2 Methods

### 2.1 Patient selection

Inclusion criteria were: male patients ≥ 5 years of age, and enrolled in HOS (i.e., met the entry criteria of a documented diagnosis of Hunter syndrome); receiving idursulfase treatment or scheduled to begin idursulfase treatment within 30 days of study enrollment; and with signed IRB/IEC-approved informed consent. Patients were not enrolled if they had received biologic or ERT products other than idursulfase or other investigational products within 30 days prior to study entry, if the patient had a life expectancy of < 2 years, or if the patient was unable to comply with the protocol.

### 2.2 Study design

Baseline values were recorded up to 30 days prior to the first idursulfase infusion in the study. The treatment period ran from Week 1 through Week 109 (approximately 2 years). Baseline and Week 1 study visits could occur on the same day provided that all selection criteria were met.

Baseline data included: medical history, physical examination, serum anti-idursulfase antibody levels (IgG, IgM, and IgE), and uGAG levels and urine creatinine. Cognitive impairment (“yes/no”) was assessed by the HOS investigator prior to or within 6 months after study entry.

Vital signs and infusion rates (start and end time points) were recorded at each weekly infusion visit. Blood and urine samples were collected every 3 months for anti-idursulfase antibody testing, and uGAG and urine creatinine measurements.

Patients were monitored for the occurrence of adverse events (AEs), and the use of concomitant medications.

### 2.3 Antibodies

Positive samples from preliminary anti-idursulfase IgG screening using the conformation-specific antibody (CSA) or isotype specific enzyme-linked immunosorbent assays (ELISA) [3] were confirmed by a corresponding isotype-specific radioimmunoprecipitation (RIP) assay [4]. Positive results of the RIP assay, were reported as positive and the titer determined by either the CSA or ELISA. Negative RIP results were reported as antibody negative. Overall antibody status referred to the anti-idursulfase IgG antibody result, and was defined as positive if one or more IgG results were found to be positive during the study.

All antibody-positive samples were analyzed for neutralizing antibodies with both an in vitro activity neutralizing antibody assay and a cell-based internalization antibody assay [7], [8].

IgG antibody status was classified into the following categories for each individual patient: 1) Antibody positive (Ab +): A patient was considered Ab + if one or more IgG antibody results were positive during the course of study. 2) Antibody negative (Ab −): A patient was Ab − if all the IgG antibody results were negative during the course of study.

For each individual patient, neutralizing antibody status was classified into one of two categories: 1) Overall neutralizing antibody status positive (NAb +): A patient was considered NAb + if one or more in vitro enzyme activity inhibition assays or one or more cell-based-internalization assays were positive during the course of study. 2) Overall neutralizing antibody status negative (NAb −): A patient was considered NAb − if all the in vitro enzyme activity inhibition assays and all the cell-based-internalization assays were negative during the course of study.

### 2.4 Safety and pharmacodynamics measurements

Urinary GAG levels, normalized to urinary creatinine concentration (to control for variations in urine flow rate) were analyzed by a central laboratory (Shire, Lexington, MA).

AEs, treatment-emergent AEs (TEAEs), serious AEs (SAEs), and IRAEs were monitored. TEAEs were defined as all AEs occurring on or after the date of first idursulfase infusion within the current study and within 30 days of the patient's last infusion. IRAEs were defined as all AEs that occurred during the infusion, or within 24 h following the infusion; and were judged as possibly or probably related to idursulfase infusion.

### 2.5 Data analyses

The change and percentage change from baseline in uGAG levels were compared between antibody groups using the Wilcoxon rank sum test. IRAE rates by antibody status groups (Ab + versus Ab −) were compared. For analysis, we conservatively assumed that any seroconversion occurring after study baseline and on or before Week 13 occurred by the date of first study infusion. To account for potentially differing follow-up times between antibody groups, the analysis was based on a negative binomial regression model. This model (model 1) was used to estimate the rate of IRAEs and the relative risk comparing positive with negative antibody status groups. Similarly, the above model was used to assess the impact of neutralizing antibody status on IRAE incidence. The negative binomial model (model 2) was used to explore the impact of adjustment for age at study enrollment (classified as < 12 years versus ≥ 12 years). Generally no imputation of missing data was performed and all analyses were considered exploratory in this observational cohort study.

## 3 Results

### 3.1 Study population

Due to difficulties identifying idursulfase ERT-naïve patients in this rare disease population, the study was modified to halt enrollment and complete the collection of data for the 26 non-naïve patients. No treatment-naïve patients were included in the study.

The characteristics of the 26 patients at baseline are shown in Table 1.

**Table 1:** Baseline characteristics by overall immune status.

Baseline characteristics	Overall (N = 26)	Ab + (n = 13)	Ab − (n = 13)	NAb + (n = 9)	NAb − (n = 17)
Age category, n (%)					
< 12 years	17 (65.4)	10 (76.9)	7 (53.8)	7 (77.8)	10 (58.8)
≥ 12 years	9 (34.6)	3 (23.1)	6 (46.2)	2 (22.2)	7 (41.2)

Age at entry, y					
Mean (SD)	12.8 (8.0)	9.3 (2.9)	16.3 (9.9)	9.5 (3.1)	14.6 (9.3)
Median	10.0	9.4	11.2	9.4	10.1
Min, Max	5.1, 35.5	5.8, 14.5	5.1, 35.5	6.2, 14.5	5.1, 35.5

Age at diagnosis, y					
Mean (SD)	4.4 (3.9)	3.5 (1.7)	5.3 (5.2)	3.3 (1.8)	5.0 (4.6)
Median	3.8	4.1	3.0	3.5	4.1
Min, Max	0.1, 20.0	0.1, 5.5	0.1, 20.0	0.1, 5.5	0.1, 20.0

Idursulfase ERT exposure before baseline, mo					
Mean (SD)	39.9 (21.5)	32.9 (17.9)	47.0 (23.0)	36.6 (19.8)	41.7 (22.7)
Median	35.8	29.1	42.6	29.5	35.9
Min, Max	6.2, 77.5	10.6, 75.7	6.2, 77.5	10.6, 75.7	6.2, 77.5

Presence of cognitive impairment^a^, n (%)					
Yes	13 (54.2)^b^	8 (61.5)	5 (38.5)	7 (53.8)	6 (46.2)
No	11 (45.8)^b^	4 (36.4)	7 (63.6)	2 (18.2)	9 (81.8)

All patients were on weekly 0.5 mg/kg intravenous idursulfase.

Overall antibody status was defined as positive if one or more IgG results were found to be positive during the study.

Ab +, antibody positive; Ab −, antibody negative; ERT, enzyme replacement therapy; NAb +, neutralizing antibody positive; NAb −, neutralizing antibody negative.

a Assessed prior to or within 6 months after entry into study.

b N = 24.

Eleven patients discontinued the study: due to investigator termination (3 out of 26), lost to follow-up (2/26), death (2/26), withdrawal of consent (1/26), other reasons (3/26). The remaining 15 patients completed the study. Median duration of idursulfase ERT prior to study enrollment was 35.8 months, and all patients had received at least 6 months of treatment with idursulfase prior to entering the study. The presence of cognitive impairment was assessed by the HOS investigator for 24 of the 26 enrolled patients. Twelve of 16 patients (75%) who were < 12 years had cognitive impairment, whereas only 1 of 8 (12.5%) patients ≥ 12 years did.

Eleven out of 26 patients were seropositive at study baseline and 2 additional patients became so between baseline and Week 13, or at the Week 13 evaluation. As antibody testing was performed at discrete time points (e.g. study baseline [up to 30 days prior to first infusion] and Week 13), the exact time of any seroconversion is unknown. Baseline data show the majority of patients < 12 years were Ab + (10/17, 59%), and the majority of patients ≥ 12 years were Ab − (6/9, 67%). This was also true for baseline neutralizing antibody status. Patients who were Ab + and NAb + at baseline were diagnosed with MPS II at a younger age (mean age at diagnosis 3.49 and 3.31 years, respectively) than those who were Ab − and NAb − (5.34 and 5.00 years, respectively). Median exposure to idursulfase at baseline was lower in the Ab + group (29.1 months) than in the Ab − group (42.6 months).

No patients seroconverted (became Ab +) after Week 13, and only 2 patients tested positive for the first time at Week 13.

Eight of 13 patients (61.5%) who had cognitive impairment were Ab + overall, whereas only 4 of 11 (36.4%) patients without impairment were Ab +. Seven of 13 patients (53.9%) who had cognitive impairment were NAb + overall, whereas only 2 of 11 (18.2%) patients without cognitive impairment were NAb +.

Four patients tested positive for idursulfase-specific IgM. None of the patient samples in this study were positive for IgE.

### 3.2 Pharmacodynamics

#### 3.2.1 uGAGs by Ab status

**Fig. 1:**
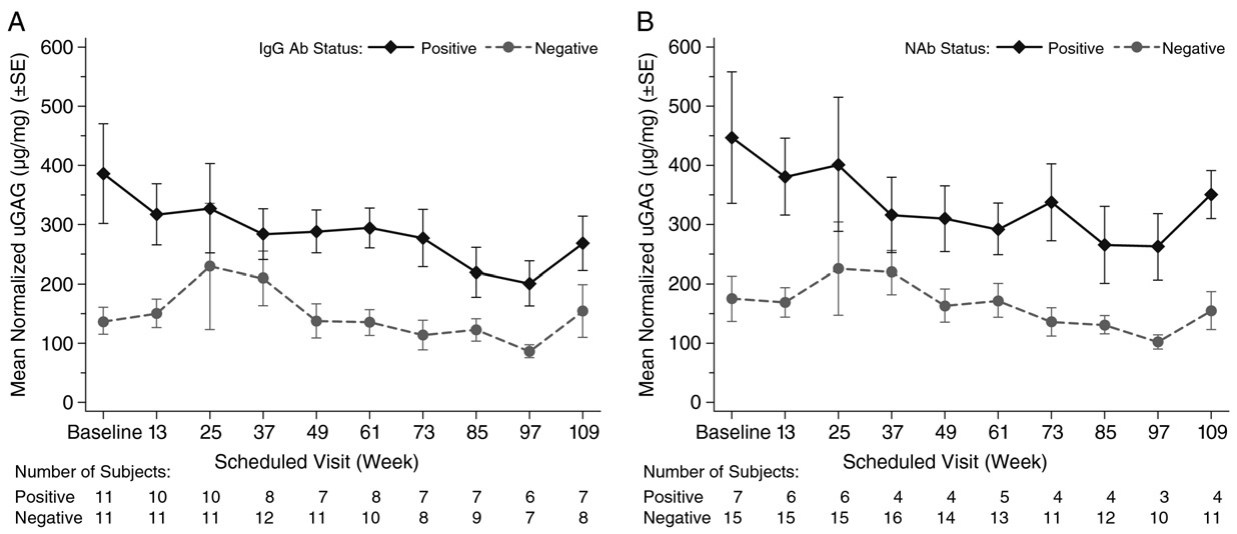
Mean normalized uGAG (μg/mg) by visit and IgG Ab status (A) and NAb status (B). Ab, antibody; IgG, immunoglobulin G; NAb, neutralizing antibodies; uGAG, urinary glycosaminoglycan.

At baseline, patients in the Ab + group had higher uGAG levels (mean 386 ± 279 μg/mg [43.7 ± 31.6 mg/mmol creatinine]) than patients in the Ab − group (mean 138 ± 76 μg/mg [15.6 ± 8.6 mg/mmol creatinine]) (Fig. 1 A).

Normal reference range for uGAG levels, adopted for this study, are ≤ 16.0 mg GAG/mmol creatinine in children aged 3–5 years, and ≤ 6.5 mg GAG/mmol creatinine in those aged > 14 years [9]. Pretreatment uGAG levels were not known because all patients in this study were on idursulfase ERT at baseline. Therefore, no assessment could be made regarding the role of antibodies on the pharmacodynamic response to idursulfase, so whether the higher baseline uGAG levels observed in Ab + patients vs Ab − patients were due to differences in therapeutic response cannot be determined. In general, uGAG levels for both Ab + and Ab − patients remained stable and none of the differences between Ab + and Ab − patients in mean change and mean % change from study baseline were statistically significant. Results for NAb were similar to those for overall antibody status (Fig. 1 B).

### 3.3 Safety

AEs were experienced by 25/26 (96.2%) patients overall. These AEs were generally mild to moderate (Table 2).

**Table 2:** Summary of TEAEs by IgG Ab status, and NAb status.

Description, n (%)	Antibody status				
	Overall	Ab +	Ab −	NAb +	NAb −
	(N = 26)	(n = 13)	(n = 13)	(n = 9)	(n = 17)
No AE	1 (3.8)	1 (7.7)	0	1 (11.1)	0
Any AE	25 (96.2)	12 (92.3)	13 (100.0)	8 (88.9)	17 (100.0)
Deaths	2 (7.7)	2 (15.4)	0	2 (22.2)	0
Discontinued due to an AE	2 (7.7)	2 (15.4)	0	2 (22.2)	0
Adverse drug reaction	9 (34.6)	5 (38.5)	4 (30.8)	4 (44.4)	5 (29.4)
SAE	16 (61.5)	9 (69.2)	7 (53.8)	8 (88.9)	8 (47.1)
Severe or life-threatening AE	12 (46.2)	7 (53.8)	5 (38.5)	7 (77.8)	5 (29.4)
IRAE	9 (34.6)	5 (38.5)	4 (30.8)	4 (44.4)	5 (29.4)

Ab, antibody; Ab +, antibody positive; Ab −, antibody negative; AE, adverse event; IRAE, infusion-related adverse event; NAb +, neutralizing antibody positive; NAb −, neutralizing antibody negative; SAE, serious adverse event; TEAE, treatment-emergent adverse event.

Sixteen patients experienced SAEs, including 2 patients who died during the study. The most common SAEs were convulsion (5/26, 19.2%) followed by lower respiratory tract infection (3/26, 11.5%). Central line infection, upper respiratory tract infection, carpal tunnel syndrome, psychiatric disorder and agitation were reported SAEs in 2 patients (7.7%) each. All of the events were considered by the investigator to be unrelated to idursulfase treatment, except for the one IRAE of central line infection. In general, Ab + and NAb + patients experienced more SAEs (69.2% and 88.9%, respectively) than Ab − and NAb − patients (53.8% and 47.1%, respectively).

Discontinuations due to AEs occurred in 2 patients, both of whom died during the study (one from pneumonia and one from respiratory failure), likely due to progression of the underlying disease. The most common AEs overall were pyrexia (14/26 patients, 54%), followed by upper respiratory tract infection (13/26, 50%), cough (11/26, 42%), ear infection, lower respiratory tract infection, and fall (9/26, 35% each). The AEs were generally similar in all antibody groups. Nine out of 26 patients overall (35%) experienced an adverse drug reaction, with adverse drug reaction defined as any TEAE event considered to be related to the study drug. Pyrexia was the most frequent adverse drug reaction (4/26, 15.4%) in all antibody groups.

At least one IRAE was experienced by 9/26 (35%) of patients. The interpretation of IRAE incidence is complicated by the fact that a total of 14/26 patients (including 7 patients at Week 1) received premedication to prevent IRAEs, and none of the patients enrolled in the study were treatment naïve. The most common IRAEs overall were pyrexia (4/26, 15%) and headache (3/26, 12%). These were the only IRAEs reported by 3 (≥ 10%) or more patients. In this small study dataset, Ab + patients appeared to have a higher risk of developing an IRAE than Ab − patients (Table 3).

**Table 3:** Comparing IRAE rates between Ab + and Ab −, NAb + and NAb −: negative binomial regression model.

Model	Independent variable	Estimated IRAE rate	RR	95% CI	*P*-value
Ab ±					
1	IgG antibody status		2.873	0.731, 11.296	0.1309
	Ab +	0.0121			
	Ab −	0.0042			
2	IgG antibody status		2.082	0.563, 7.706	0.2718
	Ab +	0.0055			
	Ab −	0.0026			
	Age at study entry		6.79	0.766, 60.225	0.0854
	< 12 Years	0.0099			
	≥ 12 Years	0.0015			

NAb ±					
1	IgG antibody status		2.976	0.788, 11.239	0.1078
	NAb +	0.0148			
	NAb −	0.0050			
2	IgG antibody status		2.264	0.661, 7.755	0.1934
	NAb +	0.0066			
	NAb −	0.0029			
	Age at study entry		6.848	0.782, 59.932	0.0822
	< 12 Years	0.0114			
	≥ 12 Years	0.0017			

The IRAE average weekly rates, RR with 95% CI and *P*-value are based on the negative binomial model. The dependent variable is the count of the number of IRAEs, and the offset variable is the natural log of the time in weeks from first study infusion to last on-study assessment (Week 109 or early termination).

Ab +, antibody positive; Ab −, antibody negative; IRAE, infusion-related adverse event; NAb +, neutralizing antibody positive, NAb −, neutralizing antibody negative; RR, relative risk.

In comparing the Ab + with the Ab − groups, Ab + patients had an almost 3-fold higher risk of an IRAE (with a Relative Risk [RR] = 2.87) (model 1 in Table 3). Inclusion of age as a variable in the model decreased the RR estimate to 2.08 (model 2 in Table 3), showing that age was an important confounder in the model.

The relationship between NAb status, age, and risk of an IRAE was similar to that observed in the Ab groups, with the RR of an IRAE being about 2-fold higher in the NAb + group after adjusting for age. However, no antibody or age group differences were statistically significant at the 5% level.

## 4 Discussion

Intravenous ERT with idursulfase is the standard treatment for patients with Hunter syndrome [10]. Several papers have described efficacy, safety, and antibody formation for idursulfase ERT in treatment-naïve patients, both in young children (up to 7.5 years) [11], [12], and patients 6 years and older [3], [4], [5].

This 2-year study assessed antibody status, pharmacodynamic effect (uGAG levels) and safety, specifically infusion-related reactions in patients receiving long-term idursulfase. The main limitation to interpretation of study results was the fact that the patients were not treatment-naïve at study entry and therefore pre-ERT assessments were not available. Other limitations were that for individual patients, the risk for IRAEs was modified by the use of premedication prior to enrollment and small sample sizes limited the ability to detect statistical differences. Premedication treatments included reduction in speed of infusion, and administration of antihistamines or steroids. The study was also relatively short, given that patients are generally receiving long term treatment. A further limitation is that genotype data for this patient population are not available for analysis.

Half of the patients (13/26) were seropositive at some point during this 2 year study, and 11/13 of these were already seropositive at baseline. The immunogenicity analysis of the original pivotal, phase 2/3 idursulfase study and 2-year extension phase similarly found 50% of patients had developed antibodies [3], [5]. In the phase 2/3 study, neutralizing antibodies were found in about one-fifth of patients (13/63, 21%), compared with 35% (9/26) found in the current study, possibly due to differing genetic make-up of the study populations [4]. Our data suggest a relationship between cognitive impairment, age, and seropositivity. Three fourths (75%) of younger patients (< 12 years) were cognitively impaired, whereas only 1 patient (12.5%) ≥ 12 years was cognitively impaired. Cognitively impaired patients were more likely to be Ab + and NAb + (61.5% and 53.9%, respectively) compared with those who were cognitively intact (36.4% and 18.2%, respectively). Ab − patients tended to be older with greater prior ERT exposure. An explanation for this may be that patients diagnosed earlier tend to have more severe disease and this may reflect a genotype that is more predictive of severe disease (such as complete deletion/large rearrangement, or nonsense mutations versus more benign missense mutations) [13]. Patients with these more serious mutations are more likely to produce a greater immune response than is seen in patients with milder mutations. For example, analyses of immunogenicity data from a study of idursulfase ERT in children 16 months to 7.5 years of age, indicated a clear association between the most severe genotype (complete deletion/large rearrangements) for MPS II and higher seroconversion rates and risk for IRAEs [12]. Likewise in Pompe disease, patients with less endogenous or functioning human acid α-glucosidase (cross-reactive immunologic material-negative: associated with nonsense genotypes and more severe disease) have been shown to have greater α-glucosidase antibody titers compared to those with more benign splice site or frameshift α-glucosidase mutations [14].

Four patients tested positive for idursulfase-specific IgM; however since IgM is typically released both early and transiently during the course of an immune response [15], and no patient was treatment naïve, IgM levels are less relevant when considering reactions to long-term treatments.

The relationship between antibody status and pharmacodynamics was analyzed by measuring uGAG levels over the duration of the study. We found that Ab − and NAb − patients had lower baseline uGAG levels at study entry than those who were Ab + and NAb +, and that this difference was maintained over the 2 year study period. Since no pretreatment data are available for this study, no assessments could be made regarding whether the observed higher baseline uGAG levels in Ab + patients were due to differences in therapeutic response or to differences in pre-treatment levels. Previous studies have demonstrated that uGAG levels in treatment-naive patients respond to treatment with idursulfase [3], [5], [11]. The majority of the decrease occurs quickly within the first few months of therapy, followed by a stable long-term maintenance phase. Given that all patients in the study had received treatment with idursulfase prior to enrollment, the largest part of any treatment effect, would have most likely occurred before study entry. Our data show that any previously attained pharmacodynamic efficacy was maintained during this study.

There were no meaningful clinical differences in the number or type of AEs observed between patients with different antibody status. This agrees with the phase 2/3 study where TEAEs and SAEs were statistically no more likely to occur in Ab + patients than in Ab − patients. AEs were experienced by virtually all patients (96%), a common finding in ERT idursulfase studies [3], [5], [11].

In this observational study, which included both pediatric and adult patients, age appeared to be an important predictor of immunogenicity and risk. Among patients < 12 years old, 59% were Ab + at baseline compared to only 33% of those ≥ 12 years. Patients who were Ab + had a 2 to 3 times greater risk of experiencing IRAEs than those who were Ab −. Age was an even greater factor with patients < 12 years old having an IRAE risk approximately 7 times that of older patients. Receiving premedication for infusion is an indicator of prior IRAE history. In this study, 7/17 (41%) of patients < 12 years of age were pre-medicated at week 1 while, none of the adolescent/adult patients (≥ 12 years) were pre-medicated. Of the 9 patients reporting IRAEs during the study, 8 were pediatric patients versus only one adult patient. Younger patients likely had more IRAEs before enrolling into the study, so it is not surprising to find younger age associated with Ab + status and more study IRAEs. Pano et al. demonstrated an association between the most severe MPS II genotypes and risk for IRAEs in Hunter syndrome children on idursulfase ERT [12]. As younger age was an indicator of more severe disease, this possibly explains its association with IRAE rates (and antibody status). Similar findings were observed for NAb status [12]. Unfortunately, the mutation status for patients in our study is not available.

IRAEs typically vary in frequency and intensity within the first 6 months of ERT, followed by a stable period with reduced need for premedication [3], [5]. For the present study, the 95% confidence intervals around the RR for IRAEs were wide, and no statistically significant differences between antibody or age groups were observed. The pivotal study and its extension, found that IRAEs were twice as likely to occur in patients who developed antibodies to idursulfase [4]. This study, where patients already had at least 6 months of treatment and 53% (14/26) of patients received premedication to prevent IRAEs, makes comparisons difficult with the pivotal study [3] where patients were treatment naïve at baseline. Nine patients in the Ab + group and 5 in the Ab − group received premedication, which may have biased the observed difference between antibody groups in IRAE rates downward toward zero.

## 5 Conclusions

Long-term treatment with idursulfase (intravenous weekly infusion at 0.5 mg/kg) was found to be safe and well tolerated for the 26 patients who enrolled in the study and had been previously treated for at least 6 months prior to study entry, and for over 2 years of follow-up for the 15 pediatric and adult MPS II patients who completed the study. The interpretation of study results is limited by the fact that patients were not treatment naive at study entry and their risk for IRAEs was modified by use of premedication both before enrollment and/or during the study. The immunogenicity profile, showing that approximately half of the patients had at least one sample positive for antibodies, was similar to that shown in previous studies. It was observed that Ab + patients had higher uGAG levels than Ab − patients, and antibodies developed more frequently in younger patients with more severe disease. Given the small sample size, a statistically significant effect of antibody status on IRAE rates or changes in uGAG levels was not demonstrated.

## Compliance with ethical standards

• Author contributions

Involved in conception and design: Arian Pano, Yongchang Qiu, David Whiteman

Analysis and interpretation of data: All authors

Drafting the article or revising it critically for important intellectual content: All authors

• Guarantor: Roberto Giugliani
• Details of funding: This study was funded by Shire (NCT00882921). Although employees of the sponsor were involved in the design, collection, analysis, interpretation, fact checking of information, and coordination and collation of comments, the content of this manuscript, the interpretation of the data, and the decision to submit the manuscript for publication in Molecular Genetics and Metabolism Reports was made by the authors independently.
• Details of ethics: This study was approved by the IRB/IEC for each investigational site and was conducted in accordance with the ethical standards of the responsible committee on human experimentation (institutional and national) and with the Declaration of Helsinki.
• Informed consent: All patients or their legally authorized guardian provided written informed consent prior to participation in this study.
• Animal rights: not applicable.

## Competing Interests and Financial Disclosures

Dr. Harmatz has received travel, honorarium, and consulting fees from BioMarin, Shire, PTC, and Alexion, and has undertaken contracted research for Armagen, Alexion-Enobia, BioMarin, FerroKin-Shire, Sanofi-Genzyme, and Shire. He has also consulted for Inventiva, Chiesi, and Telethon Institute of Genetics and Medicine, has been the recipient of educational grants from BioMarin, Sanofi-Genzyme, and Shire, and speaker bureau participant for Alexion and Sanofi-Genzyme. Dr. Giugliani has received consulting fees from, and has undertaken contracted research for Amicus, BioMarin, Sanofi-Genzyme, and Shire. In addition, he has received fees from Actelion, BioMarin, Sanofi-Genzyme, and Shire. Dr. Mendelsohn has undertaken contracted research for BioMarin, Sanofi-Genzyme, and Shire. Dr. Vellodi has no conflicts of interest or disclosures. Dr. Hendriksz has received consulting fees from Actelion, BioMarin, GlaxoSmithKline, and Sanofi-Genzyme, and has undertaken contracted research for Actelion, Amicus, BioMarin, GlaxoSmithKline, Sanofi-Genzyme, Shire, and Synageva. Dr. Vijayaraghavan has received consulting fees from BioMarin, Sanofi-Genzyme, and Shire, and has undertaken contracted research for Shire. At the time of the study Dr. Pano was an employee of Shire. Drs. Qiu, and Whiteman are employees of, and own stock in, Shire.

## Funding

This study was funded by Shire (NCT00882921), Lexington, MA USA.
